# Palmitic Acid Versus Stearic Acid: Effects of Interesterification and Intakes on Cardiometabolic Risk Markers—A Systematic Review

**DOI:** 10.3390/nu12030615

**Published:** 2020-02-26

**Authors:** Merel A. van Rooijen, Ronald P. Mensink

**Affiliations:** Department of Nutrition and Movement Sciences, NUTRIM (School of Nutrition and Translational Research in Metabolism), Maastricht University Medical Center, 6200 MD Maastricht, The Netherlands; m.vanrooijen@maastrichtuniversity.nl

**Keywords:** palmitic acid, stearic acid, positional distribution, interesterification, longer-term, postprandial, lipids, lipoproteins, cardiometabolic risk markers, coronary heart disease

## Abstract

Fats that are rich in palmitic or stearic acids can be interesterified to increase their applicability for the production of certain foods. When compared with palmitic acid, stearic acid lowers low-density lipoprotein (LDL)-cholesterol, which is a well-known risk factor for coronary heart disease (CHD), but its effects on other cardiometabolic risk markers have been studied less extensively. In addition, the positional distribution of these two fatty acids within the triacylglycerol molecule may affect their metabolic effects. The objective was to compare the longer-term and postprandial effects of (interesterified) fats that are rich in either palmitic or stearic acids on cardiometabolic risk markers in humans. Two searches in PubMed/Medline, Embase (OVID) and Cochrane Library were performed; one to identify articles that studied effects of the position of palmitic or stearic acids within the triacylglycerol molecule and one to identify articles that compared side-by-side effects of palmitic acid with those of stearic acid. The interesterification of palmitic or stearic acid-rich fats does not seem to affect fasting serum lipids and (apo) lipoproteins. However, substituting palmitic acid with stearic acid lowers LDL-cholesterol concentrations. Postprandial lipemia is attenuated if the solid fat content of a fat blend at body temperature is increased. How (the interesterification of) palmitic or stearic acid-rich fats affects other cardiometabolic risk markers needs further investigation.

## 1. Introduction

During the last decades, many studies have been carried out to gain more insight into the effects of dietary fat intake on risk markers for cardiovascular disease (CVD), such as disturbances in lipid metabolism, glucose-insulin homeostasis, the hemostatic system, or low-grade systemic inflammation. A well-accepted risk factor for coronary heart disease (CHD) is low-density lipoprotein (LDL)-cholesterol (LDL-C), which is increased by diets that are rich in saturated and *trans* fatty acids. Therefore, guidelines to prevent CHD are focused on the exchange of dietary saturated and *trans* fats for unsaturated fats [1]. However, saturated fat is a collective term for different saturated fatty acids that exert different metabolic effects. In the Western diet, palmitic acid (C16:0) and stearic acid (C18:0) are the most commonly consumed saturated fatty acids [2]. It is generally believed that palmitic acid is more cholesterol-raising than stearic acid [3,4]. However, the effects of palmitic and stearic acids on other cardiometabolic risk markers are less well established. Besides chain length of saturated fatty acids, the positional distribution of fatty acids within the triacylglycerol (TAG) molecule might also be important for their metabolic effects [5]. TAG molecules consist of a glycerol backbone to which three fatty acids are esterified. The positional distribution of these fatty acids within the TAG molecule, the so-called TAG structure, can be specified by stereospecific numbering (*sn*) as *sn*-1, *sn*-2, and *sn*-3. With interesterification, a chemical or enzymatic process used by the food industry, fatty acid positions can be exchanged within and between TAG molecules, thereby creating new TAG structures. This structure determines the physical properties of a fat, including its melting behavior, which in turn determines the suitability of the fat for the food industry; solid fats are, for instance, more suitable for baked goods and certain types of margarines than oils. Some vegetable oils, such as palm oil, contain relatively high amounts of palmitic and/or stearic acid predominantly at the outer *sn*-1 and -3 positions [6]. The interesterification of these oils increases the amounts of palmitic or stearic acids at *sn*-2, which will increase their melting points. Since no *trans* fatty acids are generated by interesterification, this process seems to be a good alternative for partially hydrogenated *trans* fats. However, the positional distribution of fatty acids might affect their metabolic fate, also because the dietary fatty acid at the *sn*-2 position is largely retained when incorporated into chylomicron TAG molecules [7]. Given that fats rich in palmitic and/or stearic acid are often used for interesterification, it is important that we thoroughly understand their impact on metabolic health. Therefore, we have systematically reviewed the current knowledge on the longer-term and postprandial effects on cardiometabolic risk markers of 1) the effect of interesterification of either palmitic acid- or stearic acid-rich fats and 2) the difference between palmitic acid- and stearic acid-rich fats. 

## 2. Methods

The databases PubMed/Medline, Embase (OVID), and Cochrane Library were searched for papers published until December, 2019. Two searches were performed; one to identify articles that studied effects of the position of palmitic acid or stearic acid on the TAG molecule and one to identify articles that compared the side-by-side effects of palmitic acid with those of stearic acid. For the effect of TAG structure, the following search strategies were used: ((interesterified[All Fields] OR "esterification"[MeSH Terms] OR "TAG structures"[All Fields] OR "triglycerides/administration and dosage"[MeSH Terms]) AND ("palmitic acid"[All Fields] OR "stearic acid"[All Fields])) for PubMed, ((triglyceride structure/ OR *triacylglycerol/ OR interesterification.mp.) AND (stearic acid/ OR palmitic acid/)) with ‘article’ as filter for Embase, and ((esterification [MeSH descriptor] OR triglycerides [MeSH descriptor with qualifier administration and dosage] OR TAG structures OR interesterified) AND (palmitic acid OR stearic acid)) in Cochrane Library. For the comparison of palmitic acid with stearic acid, the following search strategies were used: (("palmitic acid"[All Fields] OR "palmitate"[All Fields] OR "hexadecanoic acid"[All Fields] OR "C16:0"[All Fields]) AND ("stearic acid"[All Fields] OR "octadecanoic acid"[All Fields] OR "stearate"[All Fields] OR "C18:0"[All Fields])) AND "clinical study"[Publication Type] for Pubmed, (*palmitic acid/ and *stearic acid/ and human.mp) for Embase, and (palmitic acid AND stearic acid) for the Cochrane Library. 

Studies were eligible if they met the following inclusion criteria: human dietary intervention trial comparing diets or meals containing either palmitic or stearic acid mainly at *sn*-1 and -3 with diets or meals containing higher amounts of palmitic or stearic acid at the *sn*-2 position or comparing diets or meals that are rich in palmitic acid with diets or meals rich in stearic acid; diets or meals had comparable contents of saturated fatty acids (SFAs), monounsaturated fatty acids (MUFAs), and polyunsaturated fatty acids (PUFAs); subjects were ≥18 years and apparently healthy; cardiometabolic risk markers (lipids and lipoproteins, hematological markers, glucose-insulin homeostasis, endothelial function markers, and/or inflammation markers) were assessed; the articles were published in English and available as full text.

**Figure 1:**
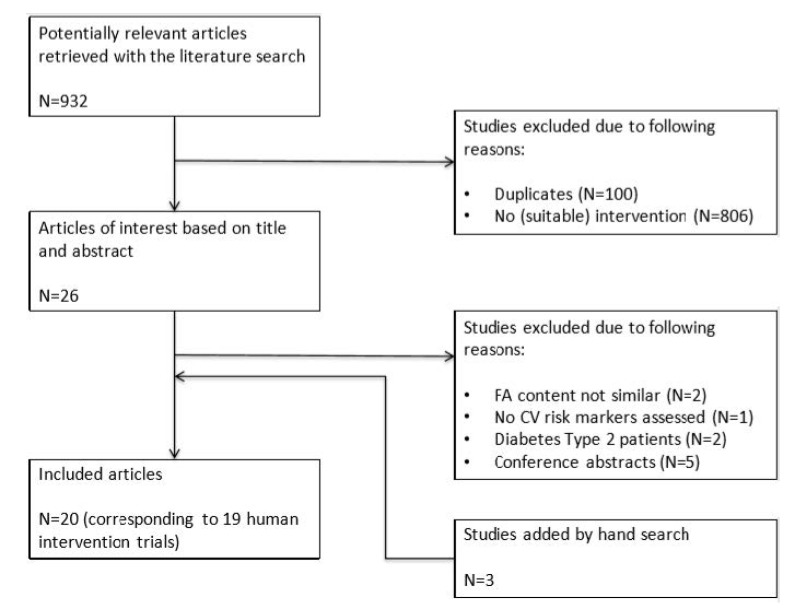
Flow chart of studies on the effects of interesterification of palmitic acid- or stearic acid-rich fats on cardiometabolic risk markers. Abbreviations: FA, fatty acid; CV, cardiovascular.

The search for the effect of the position of either palmitic or stearic acid within the TAG molecule resulted in a total of 932 records (248 from PubMed, 646 from Embase, 38 from Cochrane), of which 100 records were duplicates. Twenty-six records from the remaining 832 were considered to be of interest based on their titles and abstracts. After the screening of the full texts, two articles were excluded because the fatty-acid contents of the experimental fats were not comparable, one because no cardiometabolic risk markers were assessed, one because subjects had type 2 diabetes, and five because they were conference abstracts. The reference lists of all eligible papers were searched for additional studies, which resulted in another three articles. In the end, a total of 20 articles corresponding to 19 human intervention trials were included (Figure 1).

**Figure 2:**
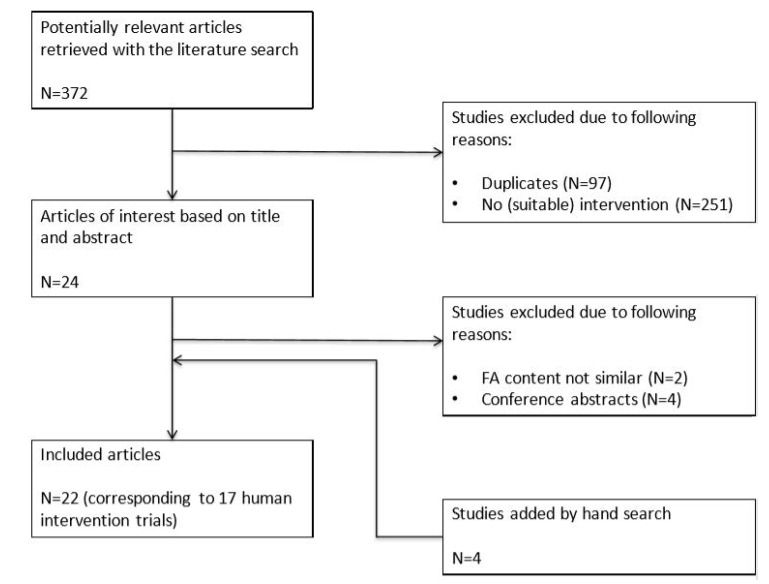
Flow chart of studies on the effects of palmitic acid versus stearic acid on cardiometabolic risk markers. Abbreviations: FA, fatty acid.

The search for palmitic acid versus stearic acid resulted in a total of 372 records (111 from PubMed/Medline, 125 from Embase, and 136 from Cochrane), of which 97 records were duplicates. Twenty-four records from the remaining 275 were considered to be of interest based on their titles and abstracts. After screening of the full texts, two articles were excluded because the experimental fats differed not only in palmitic acid and stearic acid contents, but also in other fatty acids and four other articles because they were conference abstracts. The reference lists of all eligible papers and previous reviews were searched for additional studies, which resulted in another four articles. In the end, a total of 22 articles corresponding to 17 human trials were included (Figure 2). 

## 3. Results

### 3.1. Longer-Term Effects of sn-2 Content of Palmitic Acid or Stearic Acid on Fasting Cardiometabolic Risk Markers

Six studies have compared side-by-side the effects on fasting cardiometabolic risk markers of diets with high versus low proportions of palmitic acid at the s*n*-2 position (Table A1) and two studies with high versus low proportions of stearic acid at *sn*-2 (Table A2). Table 1 summarizes the results. In seven studies, the content of palmitic or stearic acid at *sn*-2 was increased by the interesterification of experimental fats, while in one study interesterification decreased the *sn*-2 content of palmitic acid [8]. Studies examining palmitic acid-rich fats used palm oil [9,10], palm olein [11,12], butter [8], or a blend consisting mainly of coconut and palm oil [13]. Two studies have reported the solid fat content at 37 °C; in one study, both the native and interesterified palm oils were liquid [9], while interesterification increased the solid fat content of palm olein from 0 to 6% in the other study [11]. Sources for the stearic acid-rich fats were shea butter [14] and cocoa butter [15]. Interesterification of shea butter increased the solid fat content at 37 °C from 22 to 41% [14]. The melting points of native and interesterified cocoa butter were not measured, but the authors indicated that native cocoa butter was liquid at 37 °C and assumed that the solid fat content of the interesterified fat at 40.5 °C was 19% [15]. Most of the studies had used a randomized cross-over design, except for two studies that used a parallel design [8,12]. The experimental periods varied from 21 to 56 days for studies examining palmitic acid-rich fats and diets provided 1 to 11 energy percent (en%) of palmitic acid. The proportion of palmitic acids at *sn*-2 was reported in five out of seven studies and it differed between 11 and 60% of total fatty acids. The two studies examining stearic acid-rich fats had interventions periods of 18 and 21 days, and diets provided 10 and 7 en% stearic acid. One study reported proportions of stearic acid at *sn*-2, and the difference between diets was approximately 20%. 

#### 3.1.1. Lipids and (apo) Lipoproteins

The interesterification of palmitic acid-rich fats did not affect concentrations of TAG, total cholesterol (TC), LDL-C, or high-density lipoprotein (HDL)-C [8,9,10,11,12,13]. However, one study reported that men—but not women—showed a small, but statistically significant, increase in TC and LDL-C concentrations in response to the diet with a higher *sn*-2 content of palmitic acid [9]. No differences were found for non-esterified fatty acid (NEFA) [13], apolipoprotein (apo)B [8,12], apoA1 [8,11,12], and lipoprotein[a] concentrations [11,12,13]. The *sn*-2 content of stearic acid also had no effects on the concentrations of TAG [14,15], TC [14,15], LDL-C, or HDL-C [14].

#### 3.1.2. Hematological Markers

Only two studies have examined the effects of interesterification on hematological markers. No effects were found of *sn*-2 content of palmitic acid on concentrations of activated form of coagulation factor VII (FVIIa), fibrinogen, plasminogen activator inhibitor (PAI)-1 antigen, tissue plasminogen activator (tPA) antigen and its activity, and von Willebrand factor (vWF) [13], and of the stearic acid *sn*-2 content on FVIIa concentrations [14]. 

#### 3.1.3. Other Markers

The proportion of palmitic acids at *sn*-2 did not affect the concentrations of glucose [11,12,13], insulin [11,12], C-peptide [11,12], and C-reactive protein (CRP) [13]. Stearic acid *sn*-2 content also did not affect glucose and insulin concentrations [14].

### 3.2. Longer-Term Effects of Substituting Palmitic Acid with Stearic Acid on Fasting Cardiometabolic Risk Markers

Eleven studies have compared side-by-side the effects of diets that are rich in palmitic acid with those of diets rich in stearic acid on fasting cardiometabolic risk markers (Table 2 and Table A3). The palmitic acid sources used were palm oil [15,16,17,18,19,20], (interesterified) palm olein [21,22], a blend containing tripalmitin [23], and palm stearin [22]. For stearic acid-rich diets, cocoa butter [15,19,20,24], hydrogenated soybean oil [16,21], shea butter [17,18], hydrogenated canola [22], a blend containing tristearin [23], and an interesterified blend containing fully hydrogenated soybean oil [12] were used. Except for one study [12], all of the studies used a randomized cross-over design. The experimental periods varied from 18 to 56 days and diets provided 4 to 18 en% from palmitic acids or stearic acids. The exchange of palmitic acids with stearic acids between the diets varied between 1 and 15 en%. 

#### 3.2.1. Lipids and (apo) Lipoproteins

The concentrations of TAG did not differ between the diets [15,16,17,18,19,20,21,22,23,24], except in one study, where the TAG concentrations were lower after an interesterified stearic acid-rich diet [12]. However, the majority of studies found lower TC concentrations on the stearic acid-rich diet as compared with palmitic acid [15,16,17,18,19,20,23]. In five of these studies, LDL-C concentrations were also decreased [16,17,18,20,23], and in two studies the concentration of LDL-C tended to be lower on stearic acid [12,19]. Lower HDL-C concentrations on the stearic acid-rich diet were found in three studies [17,19,20], while in seven other studies, no significant differences were found [12,16,18,21,22,23,24]. No changes in concentrations of very-low density lipoprotein (VLDL)-C were reported [17,19,20]. Of the studies that measured apoB and apoA1 [12,17,19,23], one observed decreased concentrations of apoB [17] and two of apoA1 [17,19] on the stearic acid-rich diet. Lipoprotein[a] concentrations were higher on the stearic acid-rich diet in one study [25], but no differences were observed in another study [12].

#### 3.2.2. Hematological Markers

One study found decreased factor VII coagulant activity (FVIIc) on the stearic acid-rich diet when compared with palmitic acid [17]. However, FVIIc activities were not different between the diets in another study [22]. The mean platelet volume (MPV) was lower in one study [22], but no difference was observed in another study of the same group [24]. No differences between the diets were reported for other hematological markers [17,20,22,24]. In one study, various inflammation markers were measured and no significant differences were observed [20].

#### 3.2.3. Other Markers

Stearic acid decreased cholesteryl ester transfer protein (CETP) activity when compared with palmitic acid in one study [19] and a similar decrease was observed in another study, although not being significant [23]. No effects on lecithin-cholesterol acyltransferase (LCAT) activity were observed [23]. Three studies examined effects on glucose metabolism. An intravenous glucose tolerance test was performed and a comparable response in glucose and insulin was observed on both of the diets [26]. No differences were observed in the fasting concentrations of glucose [12,20], insulin [12,20], and C-peptide [12].

### 3.3. Postprandial Effects of sn-2 Content of Palmitic Acid or Stearic Acid on Cardiometabolic Risk Markers

Eight studies have compared side-by-side the postprandial effects of meals with high versus low proportions of palmitic acid at the s*n*-2 position (Table A4), and four studies with high versus low proportions of stearic acid (Table A5). Table 3 summarizes the results. Most of the studies examining palmitic acid-rich meals have used palm olein. Interesterification of palm olein not only increased the palmitic acid content at *sn*-2, but also the solid fat content at 37 °C. In one study, lard was used [27], in which interesterification decreased the palmitic acid at *sn*-2, as well as the solid fat content. Another study used a commonly consumed blend of palm stearin and palm kernel (PSt/PK) [28]. The interesterification of the PSt/PK blend increased palmitic acid at *sn*-2, but decreased the solid fat content at 37 °C. The stearic acid-rich meals consisted of structured TAG molecules with predominantly stearic and oleic acid (C18:1) [29], cocoa butter [30], shea butter [14], or canola stearin [31]. The interesterification of cocoa and shea butter increased the proportion of stearic acid at *sn*-2 and the solid fat content at 37 °C, which decreased after interesterification of canola stearin. For palmitic acid-rich meals, the total fat content of the meals varied between 40 and 75 grams, of which 12 to 30 grams originated from palmitic acid. Differences between meals in the proportion of palmitic acids at *sn*-2 varied between 17.0 and 66.8% of total fatty acids at *sn*-2. For stearic acid-rich meals, total fat content varied between 50 and 102 grams, including 17 to 30 grams of stearic acid. Two of the four studies reported the proportions of stearic acids at *sn*-2 and differences between meals were 19.7 and 25.0%. Postprandial follow-up varied between four and eight hours.

#### 3.3.1. Lipids and (apo) Lipoproteins

A lower postprandial TAG response—as indicated by the incremental area under the curve (iAUC)—was observed in one study after a meal with higher palmitic acid *sn*-2 content [32]. The same tendency was found in three other studies [27,33,34], and this was accompanied by a significant lower response in the first four hours after the meal with a higher proportion of palmitic acid at *sn*-2 in one study [34]. In contrast, one study showed an increased TAG response after a higher palmitic acid *sn*-2 content [28]. Two other studies found no differences in TAG responses [35,36]. Postprandial responses of NEFAs [7,27,32,34,35,36], TC [7,27,33,34], and HDL-, LDL- [33], VLDL-, and chylomicron- cholesterol [27,32] were comparable. ApoB48 responses were measured in one study and no effect of *sn*-2 palmitic acid content was observed [7]. For stearic acid, three studies found no changes in the total TAG responses in healthy-weight subjects [14,29,31]. An obese group was included in one of these studies, in which the TAG response was decreased after the high *sn*-2 stearic acid meal [31]. In addition, in another study, higher *sn*-2 stearic acid content decreased the TAG response in healthy-weight subjects [30]. The NEFA responses were not differently affected [14,29,31]. In addition, the responses of TC, as well as of LDL-C and HDL-C, were comparable between meals that differed in stearic acid *sn*-2 content [14,30,31]. 

#### 3.3.2. Hematological Markers

In one study, no effect of palmitic acid *sn*-2 content was observed on the FVIIa responses [33]. Interestingly, the effects of stearic acid *sn*-2 content were different between fat sources, i.e. cocoa butter with a lower stearic acid content at *sn*-2 increased FVIIa postprandial when compared with cocoa butter with a higher *sn*-2 content [30], while the amount of stearic acid at the *sn*-2 position of shea blends had no effect on FVIIa [14]. 

#### 3.3.3. Other Markers

Postprandial glucose and insulin responses after palmitic acid-rich meals were comparable [27,28,32,33,35,36,37]. However, one study found that the peak value of insulin appeared faster after the meal with higher *sn*-2 content of palmitic acid (after 60 instead of 90 minutes) [32], while another study observed lower insulin concentrations 30, 90, and 120 minutes after intake of the high *sn*-2 meal, which was accompanied by a tendency towards a lower total insulin response [33]. Furthermore, one study found lower glucose-dependent insulinotropic polypeptide (GIP) concentrations after the high *sn*-2 meal [37], while two other studies did not observe any differences [28,35]. Two studies also measured peptide YY (PYY), and no significant differences were found [28,37], although the PYY response tended to be less in women in one study [37]. Only one study examined inflammatory cytokines and the endothelial function marker E-selectin, and no differences were found [7]. Three studies examining stearic acid-rich meals measured postprandial glucose and insulin, and the responses were comparable between the meals [14,29,31]. Furthermore, white blood count (WBC), as measured in one study, was not affected [14].

### 3.4. Postprandial Effects of Substituting Palmitic Acid with Stearic Acid on Cardiometabolic Risk Markers

Six studies have compared side-by-side postprandial effects of meals high in palmitic acid with those high in stearic acid (Table 4). The fats that were added to enrich meals with palmitic acid were palm oil [38,39], palm olein [7,40], and a blend of tripalmitin with high-oleic sunflower oil (HOSO) [41]. For the stearic acid-rich meals, lard [7,38,40], hydrogenated HOSO [39], and a blend of tristearin with HOSO [41] were used. The fat content of the test meals varied between 50 and 90 grams, from which 9 to 37 grams originated from palmitic or stearic acids. The difference between palmitic and stearic acid in the meals ranged between 5 and 23 en%. Postprandial follow-up varied between four and eight hours (Table A6). 

#### 3.4.1. Lipids and (apo) Lipoproteins

In two studies, a lower TAG response after the meal rich in stearic acid was observed [7,40] and, in another study, lower TAG concentration three hours after the stearic acid-rich meal [39]. Other studies did not observe any differences [38,41,42]. The postprandial reduction in NEFAs was lower after stearic-acid intake in one study [7], but no differences were observed between the meals in two other studies [40,41]. Postprandial responses of VLDL-C, LDL-C, HDL-C, apoB, and apoA1 were measured in one study, but did not differ over time and between meals [41]. Additionally, the responses in postprandial concentrations of lipoprotein[a] [43], TC, and apoB48 [7] were not differently affected. 

#### 3.4.2. Hematological Markers

The postprandial responses of FVIIa after a meal rich in palmitic or stearic acid were comparable [39,42,44]. However, one study observed a non-significant lower response of FVIIa two to six hours after the stearic acid-rich meal with relatively stable FVIIa concentrations between four and eight hours, while FVIIa peaked six hours after palmitic acid and then declined [44]. FVIIc responses were measured in two studies. In one study, no differences between the meals were found [39]. In the other study, however, eight hours after the palmitic acid-rich meal FVIIc had almost returned to baseline, while it reached its highest value eight hours after the stearic acid-rich meal. Nevertheless, no difference was found in total FVIIc response [44]. 

#### 3.4.3. Other Markers

Postprandial responses of glucose [37,40], insulin [37,38,40], and C-peptide [37] were not differently affected. However, the secretion of GIP was lower after the intake of stearic acid-rich lard [37]. Postprandial changes in concentrations of leptin [38,40], inflammatory cytokines [7,40], E-selectin [7], and PYY [37] were comparable. In addition, changes in CETP and lipoprotein lipase (LPL) activity did not differ between the meals [41]. 

## 4. Discussion

Interesterification is widely used by the food industry to modify TAG structures of fats to change their physical characteristics and, thereby, increase their suitability for food applications without the formation of *trans* fatty acids. The saturated fatty acids within interesterified fats are predominantly palmitic acid and stearic acid. We have systematically reviewed effects of fats rich in either palmitic or stearic acid on cardiometabolic risk markers to better understand metabolic effects of interesterified fats. Focus was on the position of palmitic acid or stearic acid within the TAG molecule and on studies that have compared side-by-side palmitic acid- versus stearic acid-rich fats. 

### 4.1. Longer-Term Effects

Although the exact intakes of interesterified fats are unknown, it has been estimated that—if all trans fats would be replaced with interesterified fats—the mean daily intake in the United States would be approximately 3 en% with an upper limit of 4.8 en% [45]. The daily intakes of interesterified fats as well as the proportions of total and *sn*-2 palmitic or stearic acids differed widely between studies. However, in most studies, interesterified fat intakes were well above the estimated upper limit of 4.8 en% [45]. Still, no effects of palmitic acid or stearic acid *sn*-2 content were found. In general, metabolically healthy and relatively young subjects were studied. In the only study that included mildly hypercholesterolemic subjects, no effects of palmitic acid *sn*-2 content were also observed [10]. Furthermore, studies using stearic acid-rich fats have only been performed in men. It is known that men and women differ in CVD risk [46] and might respond differently to dietary interventions [47]. Indeed, one study observed slightly increased TC and LDL-C in men, but not in women after intake of a fat with a higher palmitic acid *sn*-2 content [9]. However, the difference between men and women was not statistically significant, but this might be explained by lack of statistical power. Little research has been done on the hemostatic system, inflammation, and glucose-insulin homeostasis, which are all involved in the pathogenesis of CVD [48,49,50]. However, the results so far do not indicate effects of diets enriched with interesterified fats on markers that are involved in these metabolic processes.

Since the use of interesterified fats might increase stearic and/or palmitic acid intakes, we need to thoroughly understand their metabolic effects. The daily intakes of palmitic and stearic acids in the United States are approximately 6 en% and 3 en%, respectively [51]. It is well known that stearic acid lowers concentrations of TC, LDL-C, and HDL-C when compared with palmitic acid [52]. Indeed, the majority of studies showed decreased TC and LDL-C concentrations on the stearic acid-rich diet [16,17,18,23]. In three studies, lower HDL-C concentrations were observed [17,19,20]. Only one out of four studies observed a statistically significant decrease in apoB100 concentrations on the stearic acid-rich diet [17]. However, previous meta-analyses found lower apoB concentrations on stearic acid as compared with palmitic acid [3] and a non-significant increase in apoB when carbohydrates were replaced with palmitic acid but not when replaced with stearic acid [4]. TAG concentrations were comparable between diets, which might suggest that the number of VLDL particles was unchanged. Therefore, it is of interest to examine whether stearic acid induces a shift towards smaller and denser LDL particles. Furthermore, two of the four studies found decreased apoA1 concentrations [17,19]. It is uncertain whether this is associated with less (pre-β) HDL particles, since one HDL particle can contain up to four A1 apolipoproteins [53]. As apoA1 is involved in ATP-binding cassette transporter (ABC) A1-mediated cholesterol efflux from peripheral cells to pre-β-HDL particles, it is of interest to examine whether these decreased apoA1 concentrations result in impaired reverse cholesterol transport. Only a few studies examined effects on hematological markers. The platelet volume decreased when minimally 5 en% palmitic acid was exchanged for stearic acid [22]. Total platelet count was not affected, which suggests smaller platelets that are considered to be less active than larger ones [54]. In addition, FVIIc activity decreased when 14 en% palmitic acid was exchanged for stearic acid [17], but not when 5 en% was exchanged [22]. Furthermore, the first study used shea butter, while the latter used hydrogenated canola oil. It has been suggested that the effects of shea butter may be due to its non-glyceride components instead of its stearic acid content [22]. Hematological markers that were related to fibrinolysis were not affected [17,22]. Remarkably, only one of the longer-term studies included in this review has addressed the effects of palmitic and stearic acids on inflammation [20], and only two studies examined fasting glucose and insulin concentrations [12,20]. In these studies, no differences were observed, but more research is needed to confirm these results. 

### 4.2. Postprandial Effects

The postprandial TAG responses are highly dynamic and they depend on many factors. For example, gender, age, and obesity are known to affect postprandial lipemia [55]. Indeed, the studies that included obese subjects observed higher postprandial TAG responses in obese as compared with healthy-weight subjects [31,38]. In addition, one study observed lower postprandial TAG concentrations in premenopausal women than in men [7]. Normally, TAG concentrations in the blood peak three to five hours after the meal and then return to baseline within six to eight hours [56]. The studies included in this review differed in postprandial follow-up, ranging between four and eight hours. Since not only the peak value of TAG after a meal, but also the time to return to fasting TAG concentrations (the duration of lipemia) is positively related to CVD [55,56], it might be important to follow-up for at least six hours to gain more insights in both peak values and duration of lipemia. In addition, during the day, people generally consume another meal after four to six hours. However, none of the studies included a so-called second meal challenge. Introducing a second fat-rich meal four to six hours after the first meal has been shown to induce the release of chylomicrons that contain fatty acids from the previous meal [57]. Therefore, the composition of the previous meal might affect meal effects. In addition, postprandial impairment of endothelium-dependent vasodilation and oxidative stress are most marked after a second fat-containing meal [58]. Conflicting results have been reported on postprandial TAG responses of native and interesterified palmitic or stearic acid-rich fats. This discrepancy might be explained by the characteristics of the fats used, in particular the solid fat content at 37 °C. In most studies, the solid fat content increased if the proportion of palmitic acid or stearic acid at *sn*-2 increased. However, in one study, solid fat content was lower for the fat blend high in palmitic acid at *sn*-2 and the results of this study were opposite to those of other studies, e.g. higher TAG response after the fat with higher *sn*-2 palmitic acid content [28]. It has been suggested that the solid fat content at body temperature, rather than *sn*-2 palmitic or stearic acid content, determines the postprandial TAG response [5]. It is hypothesized that a high solid fat content at 37 °C, which is often due to tristearin (SSS) or tripalmitin (PPP) TAG species, impairs micelle formation [14] and reduces accessibility for pancreatic lipase [31], thereby decreasing the rate of absorption by the enterocyte. The FVIIa responses seem to be related to postprandial lipemia, e.g. attenuated lipemia is associated with decreased FVIIa responses [33]. Although no changes in glucose and insulin responses were shown between fats differing in *sn*-2 palmitic or stearic acid content, the results on postprandial release of gut hormone GIP were less clear. GIP induces insulin secretion and it is released when fatty acids and/or carbohydrates enter the small intestine [59]. GIP has only been measured in studies investigating the *sn*-2 position of palmitic acid [28,37], and the results differed between these two studies. Palm oil increased GIP more than interesterified palm oil [37], while no difference was observed after the native and interesterified blend of palm stearin and palm kernel [28]. It is likely that this is due to the difference in physical characteristics of the control fats used; fats liquid at body temperature, such as high oleic sunflower oil and palm oil, increase GIP more than fats with solids at body temperature, such as interesterified palm oil and lard [37]. The effects on GIP were possibly attenuated since both the native and interesterified blends of palm stearin and palm kernel were partly solid at body temperature [28]. The only study that has measured the effects of positional distribution within the TAG molecules on postprandial inflammatory cytokines and E-selectin observed no effects of *sn*-2 palmitic acid content in a meal [7]. Substituting palmitic with stearic acid does not seem to affect the postprandial responses of lipids and (apo) lipoproteins, although two studies observed a lower TAG response after lard when compared with palm olein [40]. However, it is uncertain if this difference is due to the exchange between palmitic and stearic acid or due to differences in *sn*-2 content of palmitic acid and subsequently physical characteristics; lard has a higher solid fat content at 37 °C. The postprandial effects on hematological markers, glucose-insulin homeostasis, and inflammation require further attention, but, so far, the results do not indicate clear differences between palmitic and stearic acids. 

## 5. Conclusions

Interesterification of palmitic acid- or stearic acid-rich fats does not seem to affect fasting serum lipids and (apo) lipoproteins. On the other hand, stearic acid decreases the LDL- and HDL-cholesterol concentrations when compared with palmitic acid. In addition, postprandial lipemia is attenuated if the changes in palmitic acid or stearic acid *sn*-2 contents increase the solid fat content of the blend at body temperature. No evidence was found that solely substituting palmitic acid with stearic acid affected postprandial lipemia. However, there is a need to further examine the fasting and postprandial effects of (interesterification of) palmitic acid- and stearic acid-rich fats on the hemostatic system, inflammation, and glucose-insulin homeostasis, as well as on emerging cardiometabolic risk markers, such as cholesterol efflux capacity and lipoprotein particle size. In addition, it would be of interest for future studies to specifically examine populations that have a higher risk for CVD, such as elderly or people with obesity, and to examine sex differences. 

## Figures and Tables

**Table 1:** Summary of studies examining the longer-term effects of substituting fats low in palmitic acid (C16:0) or stearic acid (C18:0) *sn*-2 contents with fats high in C16:0 or C18:0 *sn*-2 contents, respectively.

Fasted Lipids and lipoproteins	High vs low C16:0 *sn*-2	High vs low C18:0 *sn*-2	Hematological markers	High vs low C16:0 *sn*-2	High vs low C18:0 *sn*-2	Other markers	High vs low C16:0 *sn*-2	High vs low C18:0 *sn*-2
Triacyl- glycerol	0 ↓ 6 = 0 ↑	0 ↓ 2 = 0 ↑	FVIIa	0 ↓ 1 = 0 ↑	0 ↓ 1 = 0 ↑	Glucose	0 ↓ 3 = 0 ↑	0 ↓ 1 = 0 ↑
Non-esterified fatty acids	0 ↓ 1 = 0 ↑	NA	Fibrinogen	0 ↓ 1 = 0 ↑	NA	Insulin	0 ↓ 2 = 0 ↑	0 ↓ 1 = 0 ↑
Total cholesterol	0 ↓ 6 = * 0 ↑	0 ↓ 2 = 0 ↑	PAI-1	0 ↓ 1 = 0 ↑	NA	C-peptide	0 ↓ 2 = 0 ↑	NA
LDL- cholesterol	0 ↓ 6 = * 0 ↑	0 ↓ 1 = 0 ↑	tPA	0 ↓ 1 = 0 ↑	NA	C-reactive protein	0 ↓ 1 = 0 ↑	NA
HDL- cholesterol	0 ↓ 6 = 0 ↑	0 ↓ 1 = 0 ↑	vWF	0 ↓ 1 = 0 ↑	NA			
ApoB	0 ↓ 3 = 0 ↑	NA						
ApoA1	0 ↓ 3 = 0 ↑	NA						
Lp[a]	0 ↓ 3 = 0 ↑	NA						

Markers are significantly lower (↓), higher (↑) or not significantly different (=) after intake of fats high in C16:0 *sn*-2 or C18:0 *sn*-2 contents compared with fats low in C16:0 *sn*-2 or C18:0 *sn*-2 contents respectively. *=In men, total and LDL cholesterol concentrations were slightly increased (0.10 mmol/L and 0.08 mmol/L respectively) on the diet with higher C16:0 *sn*-2 [9]. Abbreviations: apo, apolipoprotein; FVIIa, activated factor VII; HDL, high-density lipoprotein; LDL, low-density lipoprotein; Lp[a], lipoprotein (a); NA, not available; PAI, plasminogen activator inhibitor; tPA, tissue plasminogen activator; vWF, von Willebrand Factor.

**Table 2:** Summary of studies examining the longer-term effects of substituting fats high in palmitic acid (C16:0) with fats high in stearic acid (C18:0).

Fasted Lipids and lipoproteins	C18:0 vs C16:0	Hematological markers	C18:0 vs C16:0		C18:0 vs C16:0	Other markers	C18:0 vs C16:0
Triacyl- glycerol	1 ↓ 10 = 0 ↑	FVIIc	1 ↓ 1 = 0 ↑	Fibrinogen	0 ↓ 1 = 0 ↑	CETP activity	1 ↓ 1 = 0 ↑
Total cholesterol	7 ↓ 4 = 0 ↑	Mean platelet volume	1 ↓ 1 = 0 ↑	Plasminogen	0 ↓ 1 = 0 ↑	LCAT activity	0 ↓ 1 = 0 ↑
VLDL- cholesterol	0 ↓ 4 = 0 ↑	PAI-1 activity	0 ↓ 1 = 0 ↑	White blood cells	0 ↓ 2 = 0 ↑	Glucose	0 ↓ 2 = 0 ↑
LDL- cholesterol	5 ↓ 5 = 0 ↑	PAI-1 antigen	0 ↓ 1 = 0 ↑	Red blood cells	0 ↓ 2 = 0 ↑	Insulin	0 ↓ 2 = 0 ↑
HDL- cholesterol	3 ↓ 7 = 0 ↑	tPA activity	0 ↓ 1 = 0 ↑	Hemoglobin	0 ↓ 2 = 0 ↑	C-peptide	0 ↓ 1 = 0 ↑
ApoB	1 ↓ 4 = 0 ↑	tPA antigen	0 ↓ 1 = 0 ↑	Platelets	0 ↓ 2 = 0 ↑	Various inflammation markers	0 ↓ 1 = 0 ↑
ApoA1	2 ↓ 3 = 0 ↑	EFA	0 ↓ 1 = 0 ↑	Antithrombin III	0 ↓ 1 = 0 ↑		
Lp[a]	0 ↓ 1 = 1 ↑	Thrombomodulin	0 ↓ 1 = 0 ↑	PTT	0 ↓ 1 = 0 ↑		
		Prothrombin time	0 ↓ 1 = 0 ↑	APTT	0 ↓ 1 = 0 ↑		

Markers are significantly lower (↓), higher (↑) or not significantly different (=) after intake of fats high in C18:0 compared with fats high in C16:0. Abbreviations: apo, apolipoprotein; APTT, activated partial thromboplastin time; CETP, cholesteryl ester transfer protein; EFA, euglobulin fibrinolytic activity; FVIIc, Factor VII coagulant activity; LCAT, lecithin-cholesterol acyltransferase; LDL, low-density lipoprotein; Lp[a], lipoprotein (a); HDL, high-density lipoprotein; PAI, plasminogen activator inhibitor; PTT, partial thromboplastin time; tPA, tissue plasminogen activator, VLDL, very-low density lipoprotein.

**Table 3:** Summary of studies examining the postprandial effects of substituting fats low in *sn*-2 palmitic acid (C16:0) or stearic acid (C18:0) contents with fats high in *sn*-2 C16:0 or C18:0 contents respectively.

Postprandial Lipids and lipoproteins	High vs low C16:0 *sn*-2	High vs low C18:0 *sn*-2	Hemato-logical markers	High vs low C16:0 *sn*-2	High vs low C18:0 *sn*-2	Other markers	High vs low C16:0 *sn*-2	High vs low C18:0 *sn*-2
Triacylglycerol	1 ↓ 6 = 1 ↑	2 ↓ 3 = 0 ↑	FVIIa	0 ↓ 1 = 0 ↑	1 ↓ 1 = 0 ↑	Glucose	0 ↓ 7 = 0 ↑	0 ↓ 3 = 0 ↑
Non-esterified fatty acids	0 ↓ 6 = 0 ↑	0 ↓ 3 = 0 ↑	White blood cells	0 ↓ 1 = 0 ↑	0 ↓ 1 = 0 ↑	Insulin	0 ↓ 7 = 0 ↑	0 ↓ 3 = 0 ↑
Total cholesterol	0 ↓ 4 = 0 ↑	0 ↓ 3 = 0 ↑				C-peptide	0 ↓ 1 = 0 ↑	NA
VLDL-cholesterol	0 ↓ 2 = 0 ↑	NA				GIP	1 ↓ 2 = 0 ↑	NA
LDL-cholesterol	0 ↓ 1 = 0 ↑	0 ↓ 3 = 0 ↑				Peptide YY	0 ↓ 2 = 0 ↑	NA
HDL-cholesterol	0 ↓ 1 = 0 ↑	0 ↓ 2 = 0 ↑				IL-6	0 ↓ 1 = 0 ↑	NA
Chylomicron- cholesterol	0 ↓ 2 = 0 ↑	NA				IL-8	0 ↓ 1 = 0 ↑	NA
ApoB48	0 ↓ 1 = 0 ↑	NA				TNF-α	0 ↓ 1 = 0 ↑	NA
						E-selectin	0 ↓ 1 = 0 ↑	NA

Markers are significantly lower (↓), higher (↑) or not significantly different (=) after intake of fats high in C16:0 *sn*-2 or C18:0 *sn*-2 contents compared with fats low in C16:0 *sn*-2 or C18:0 *sn*-2 contents respectively. Abbreviations: apo, apolipoprotein; FVIIa, activated factor VII; GIP, glucose-dependent insulinotropic polypeptide; HDL, high-density lipoprotein; IL, interleukin; LDL, low-density lipoprotein; TNF, tumor necrosis factor; VLDL, very-low density lipoprotein.

**Table 4:** Summary of studies examining the postprandial effects of substituting fats high in palmitic acid (C16:0) with fats high in stearic acid (C18:0).

Postprandial Lipids and lipoproteins	C18:0 vs C16:0	Hematological markers	C18:0 vs C16:0	Other markers	C18:0 vs C16:0
Triacylglycerol	1 ↓ 4 = 0 ↑	FVIIa	0 ↓ 3 = 0 ↑	Glucose	0 ↓ 1 = 0 ↑
Non-esterified fatty acids	0 ↓ 2 = 0 ↑	FVIIc	0 ↓ 2 = 0 ↑	Insulin	0 ↓ 2 = 0 ↑
Total cholesterol	0 ↓ 1 = 0 ↑	PAI-1 antigen	0 ↓ 1 = 0 ↑	GIP	1 ↓ 0 = 0 ↑
VLDL-cholesterol	0 ↓ 1 = 0 ↑	tPA activity	0 ↓ 1 = 0 ↑	Peptide YY	0 ↓ 1 = 0 ↑
LDL-cholesterol	0 ↓ 1 = 0 ↑			Leptin	0 ↓ 1 = 0 ↑
HDL-cholesterol	0 ↓ 1 = 0 ↑			CETP activity	0 ↓ 1 = 0 ↑
ApoB	0 ↓ 1 = 0 ↑			LPL activity	0 ↓ 1 = 0 ↑
ApoA1	0 ↓ 1 = 0 ↑			IL-6	0 ↓ 1 = 0 ↑
Lp[a]	0 ↓ 1 = 0 ↑			TNF-α	0 ↓ 1 = 0 ↑
				IL-1β	0 ↓ 1 = 0 ↑

Markers are significantly lower (↓), higher (↑) or not significantly different (=) after intake of fats high in C18:0 compared with fats high in C16:0. Abbreviations: apo, apolipoprotein; CETP, cholesteryl ester transfer protein; FVIIa, activated factor VII; FVIIc, Factor VII coagulant activity; GIP, glucose-dependent insulinotropic polypeptide; HDL, high-density lipoprotein; IL, interleukin; LDL, low-density lipoprotein; Lp[a], lipoprotein (a); LPL, lipoprotein lipase; PAI, plasminogen activator inhibitor; TNF, tumor necrosis factor; tPA, tissue plasminogen activator; VLDL, very-low density lipoprotein.

## Appendix A

**Table A1:** Longer-term effects of substituting fats low in *sn*-2 palmitic acid (C16:0) contents with fats high in *sn*-2 C16:0 contents on fasting cardiometabolic risk. markers.

First author, Year of publication	Study population, Age, BMI	Duration intervention periods, Study design	Total fat (en%)	C16:0 (en%)	Source Low *sn*-2 High *sn*-2	C16:0 *sn*-2 in fat blends (% ^1^)	Solid fat at 37 °C (%)	Lipids and lipoproteins	Hematological markers	Other markers
Nestel, 1995 [10]	27 men (mildly hyperchol ^2^) 49 ± 8 y 26.3 ± 2.5 kg/m^2^	21 days Crossover (no WO)	31	6.7	Palm oil IE palm oil	8.7 24.7 wt%	NR	TAG = TC = LDL-C = HDL-C =		
Zock, 1995 [9]	23 men 37 women ^3^ 29 (19–67) y 22.9 (18.1–30.9) kg/m^2^	21 days Crossover (no WO)	40	11	Control and IE blend of palm oil blended with sunflower oil	6.4 66.9 wt%	0 0	TAG = TC = ^4^ LDL-C = ^4^ HDL-C =		
Meijer, 1997 [13]	30 men 30 women ± 35.5 y ± 23.8 kg/m^2^	21 days Crossover ^5^ (no WO)	34	1 or 2^5^	Control and IE blend that consisted mainly of coconut and palm oils blended with soybean oil	7.1 18.0 wt%	NR	TAG = NEFA = TC = LDL-C = HDL-C = Lp[a] =	FVIIa = Fibrinogen = PAI-1 antigen = tPA antigen = tPA activity = vWF =	Glucose = CRP =
Christophe, 2000 [8]	32 men 23–53 y 18.1–23.5 kg/m^2^	28 days Parallel	NR ± 131 g	NR ± 5 g	IE butter Butter	NR	NR	TAG = TC = LDL-C= HDL-C= ApoB = ApoA1 =		
Filippou, 2014 [11]	10 men 31 women ± 29.1 y ± 23.0 kg/m^2^	42 days Crossover (no WO)	27	9	Palm olein IE palm olein	9.8 45.9 mol%	0 5.9	TAG = TC = LDL-C = HDL-C = ApoB = ApoA1 = Lp[a] =		Glucose = Insulin = C-peptide =
Ng, 2018 [12]	64 women 21 men 20–60 y 21–30 kg/m^2^	56 days Parallel	35	7	Palm olein CIE palm olein	11.1 32.4 wt%	NR	TAG = TC = LDL-C = HDL-C = ApoB = ApoA1 = Lp[a] =		Glucose = Insulin = C-peptide =

Markers are significantly lower (↓), higher (↑) or not significantly different (=) after intake of fats high in C16:0 *sn*-2 contents compared with fats low in C16:0 *sn*-2 contents. ^1^=% of total fatty acids at sn-2. ^2^=Subjects were mildly hypercholesterolemic (Average total cholesterol: 6.00 ± 0.78 mmol/L) [10]. ^3^=Pre- and postmenopausal women were included; however, study was designed in such a way that menstrual cycle or use of oral contraceptives should not have influenced results [9]. ^4^=In men, total and LDL cholesterol concentrations were slightly increased (0.10 mmol/L and 0.08 mmol/L respectively) on the diet with higher C16:0 sn-2 [9]. ^5^=Subjects were divided into two parallel groups that were assigned to a diet with either 4 or 8 en% of the blends. Of the 60 subjects in total, 32 (16 men and 16 female) subjects followed the 4 en% diet (age ± 33 years, BMI: ± 24.1 kg/m^2^) and 28 (14 men and 14 female) subjects the 8 en% diet (age ± 38 years, BMI ± 23.4 kg/m^2^). The blends provided 1 and 2 en% palmitic acid in the 4 and 8 en% diet respectively, total amount of palmitic acid in the diets was not reported [13]. Abbreviations: apo, apolipoprotein; CIE, chemically interesterified; CRP, C-reactive protein; en%, % of total energy; FVIIa, activated factor VII; HDL-C, high-density lipoprotein cholesterol; IE, interesterified; Lp[a], lipoprotein [a]; LDL-C, low-density lipoprotein cholesterol; NEFA, non-esterified fatty acids; NR, not reported; PAI, plasminogen activator inhibitor; *sn*, stereospecific numbering; TAG, triacylglycerol; TC, total cholesterol; tPA, tissue plasminogen activator; vWF, von Willebrand Factor; WO, wash out period; wt, weight; y, year.

**Table A2:** Longer-term effects of substituting fats low in *sn*-2 stearic acid (C18:0) contents with fats high in *sn*-2 C18:0 contents on fasting cardiometabolic risk markers.

First author, Year of publication	Study population, Age, BMI	Duration intervention periods, Study design	Total fat (en%)	C18:0 (en%)	Source Low *sn*-2 High *sn*-2	C18:0 *sn*-2 in fat blends (% ^1^)	Solid fat at 37 °C (%)	Lipids and lipoproteins	Hematological markers	Other markers
Grande, 1970 [15]	32 men 40–65 y NR	18 days Latin-square	34	10	Native or IE cocoa butter ^2^ blended with safflower oil	NR	NR ^3^	TAG = TC =		
Berry, 2007 [14]	16 men 26.8 ± 8.0 y 23.7 ± 3.7 kg/m^2^	21 days Crossover	30 g test fat ^4^	7^4^	Native or IE shea butter blended with sunflower oil	3.1 22.8 mol%	22 41	TAG = TC = LDL-C = HDL-C =	FVIIa =	Glucose = Insulin =

Markers are significantly lower (↓), higher (↑) or not significantly different (=) after intake of fats high in C18:0 *sn*-2 contents compared with fats low in C18:0 *sn*-2 contents. ^1^=% of total fatty acids at *sn*-2 ^2^=the interesterified cocoa butter was a mix of palm oil, totally hydrogenated soybean oil, and olive oil which matched the fatty acid composition of native cocoa butter [15]. ^3^=Melting points of the blends were not measured. Authors reported that native cocoa butter is normally liquid at 37 °C, while they calculated that IE cocoa butter should have 19% solid fat content at 40.5 °C [15].^4^=Total daily intake of total fat and stearic acid was not reported. Diets provided 30 grams of test fat and an additional 7 en% (15 grams) of C18:0 per day [14]. Abbreviations: en%, % of total energy; FVIIa, activated factor VII; HDL-C, high-density lipoprotein cholesterol; IE, interesterified; LDL-C, low-density lipoprotein cholesterol; NR, not reported; *sn*, stereospecific numbering; TAG, triacylglycerol; TC, total cholesterol; y, year.

**Table A3:** Longer-term effects of substituting fats high in palmitic acid (C16:0) with fats high in stearic acid (C18:0) on fasting cardiometabolic risk markers.

First author, Year of publication	Study population, Age, BMI	Duration intervention period, Study design	Total fat (en%)	C16:0 C18:0 (en%)	Difference between diets C16:0 C18:0 (en%)	Main source C16:0 C18:0	Lipids and lipoproteins	Hematological markers	Other markers
Grande, 1970 [15]	32 men 40–65 y NR	18 days Latin-square	34	15 10	6 8	Palm oil Cocoa butter	TAG = TC ↓		
Bonanome, 1988 [16]	11 men 64 ± 4.0 y 24 ± 1.7 kg/m^2^	21 days Cross-over (no WO)	40	18 17	15	Palm oil Hydrogenated soybean oil	TAG = TC ↓ VLDL-C = LDL-C ↓ HDL-C =		
Tholstrup, 1994 [17] + 1995 [25]	15 men 24.9 (22–30) y 23.2 (20.4–26.4) kg/m^2^	21 days Cross-over	40	16 ^1^ 14	14	Palm oil Shea butter	TAG = TC ↓ VLDL-C = LDL-C ↓ HDL-C ↓ ApoB ↓ ApoA1 ↓ Lp[a] ↑	FVIIc ↓ PAI-1 activity = PAI-1 antigen = tPA activity = tPA antigen = EFA =	
Dougherty, 1995 [18]	10 men 37.4 ± 6.6 y 25.2 ± 2.5 kg/m^2^	40 days Cross-over (no WO)	27-29	7	5 6	Palm oil Shea butter	TAG = TC ↓ LDL-C ↓ HDL-C =		
Schwab, 1996 [19] + 1997 [26]	12 women ^2^ (premenopausal) 23.5 ± 3.1 y 22.1 ± 2.4 kg/m^2^	28 days Cross-over	37	12 7	3 5	Palm oil, butter Cocoa butter	TAG = NEFA =^2^ TC ↓ VLDL-C = LDL-C = HDL-C ↓ ApoB = ApoA1 ↓		CETP activity ↓ Glucose = ^2^ Insulin = ^2^
Nestel, 1998 [21]	15 subjects (mildly hyperchol men and women ^3^) 51 ± 7 y 26.2 ± 3.9 kg/m^2^	35 days Cross-over (no WO)	41-42	8 ^4^	±5	Palm olein Fully hydrogenated soybean oil	TAG = TC = LDL-C = HDL-C =		
Snook, 1999 [23]	16 women (premenopausal) 28 ± 6 y NR	35 days 3x3 cross-over	40	13	10 11	Tripalmitin Tristearin	TAG = TC ↓ LDL-C ↓ HDL-C = ApoB = ApoA1 =		CETP activity = LCAT activity =
Kelly, 2001 [22]	13 men 35 ± 12 y 26 ± 3.3 kg/m^2^	28 days Cross-over	27–28	8 7	6 5	Palm stearin and/or palm olein Hydrogenated canola	TAG = TC = LDL-C = HDL-C =	FVIIc = MPV ↓ Fibrinogen = Plasminogen = WBC = RBC = Hb = PLT = APTT = ATIII =	
Kelly, 2002 [24]	9 men 39 ± 10 y 25 ± 2.5 kg/m^2^	21 days Cross-over	28–29	7 4	1 2	Potato crisps, shortbread biscuits, muesli bars Milk chocolate	TAG = TC = LDL-C = HDL-C =	MPV = WBC = RBC = Hb = PLT =	
Ng, 2018 [12]	64 women 21 men 20–60y 21–30 kg/m^2^	56 days Parallel	35	7 8	5 7	IE Palm olein IE hydrogenated soybean oil	TAG ↓ TC = LDL-C = HDL-C = ApoB = ApoA1 = Lp[a] =		Glucose = Insulin = C-peptide =
Meng, 2019 [20]	20 postmenopausal women (mildly hyperchol ^5^) 64 ± 7 y 26.4 ± 3.4 kg/m^2^	35 days Cross-over	30	14 10	8 9	Palm oil Cocoa butter	TAG = TC ↓ VLDL-C = LDL-C ↓ HDL-C ↓ ApoB = ApoA1 = Lp[a] =	PT = PTT =	Glucose = Insulin = CRP = TNF-a = IL-6 = SAA-1 = sICAM-1 = sICAM-3 = sVCAM-1 = E-selectin = P-selectin = Thrombomodulin =

Markers are significantly lower (↓), higher (↑) or not significantly different (=) after intake of fats high in C18:0 compared with fats high in C16:0. ^1^Total dietary intake of C16:0 and C18:0 was not reported, values represent intakes from the blends. Blends provided 90% of total fat intake [17]. ^2^The measurements of glucose, insulin, and non-esterified fatty acids were performed in a sub study with 8 of the 12 participants. Glucose and insulin were assessed with an intravenous glucose tolerance test and due to technical reasons the results of only 6 subjects were available on both diets [26]. ^3^Number of men and women that completed the study was not reported but 12 men and 8 women were screened. Not defined if women were pre- or postmenopausal. Subjects were mildly hypercholesterolemic (Average total cholesterol: 6.13 ± 0.80 mmol/L) [21]. ^4^Total dietary intake of C16:0 and C18:0 was not reported, values represent intakes from the blends. Blends provided 55% of total fat intake [21]. ^5^Mildly hypercholesterolemic based on LDL-cholesterol concentrations (Average LDL-cholesterol: 3.5 ± 0.7 mmol/L, total cholesterol: 5.6 ± 0.8 mmol/L) [20]. Abbreviations: apo, apolipoprotein; APTT, activated partial thromboplastin time; ATIII, antithrombin III; CETP, cholesteryl ester transfer protein; CRP, C-reactive protein; EFA, euglobulin fibrinolytic activity; en%, % of total energy; FVIIc, Factor VII coagulant activity; HDL-C, high-density lipoprotein cholesterol; Hb, hemoglobin; HOSO, high oleic acid sunflower oil; IE, interesterified; IL, interleukin; LCAT, lecithin-cholesterol acyltransferase; Lp[a], lipoprotein [a]; LDL-C, low-density lipoprotein cholesterol; MPV, mean platelet volume; NR, not reported; PAI, plasminogen activator inhibitor; PLT, platelets; PT, prothrombin time; PTT, partial thromboplastin time; RBC, red blood cells; SAA, serum amyloid A; sICAM, soluble intercellular adhesion molecule; *sn*, stereospecific numbering; sVCAM, soluble vascular cell adhesion molecule; TAG, triacylglycerol; TC, total cholesterol; TNF, tumor necrosis factor; tPA, tissue plasminogen activator; VLDL, very low-density lipoprotein; WBC, white blood cells; WO, wash out period; wt, weight; y, year.

**Table A4:** Postprandial effects of substituting fats low in palmitic acid (C16:0) *sn*-2 contents with fats high in C16:0 *sn*-2 contents on cardiometabolic risk markers.

First author, Year of publication	Population, Age, BMI, Follow-up	Total energy (kcal)	Total fat in grams (en%)	C16:0 content in grams (en%)	Source Low *sn*-2High *sn*-2	C16:0 *sn*-2 in fat blends (% ^1^)	Solid fat at 37 °C (%)	Lipids and lipoproteins	Hematological markers	Other markers	
Zampelas, 1994 [35]	16 men 24.8±2.6 y 22.7±2.4 kg/m^2^ 6 h	662	40 (54 en%)	12 (16 en%)	Palm olein IE blend of palm stearine with sunflower oil	5.9 72.7 wt%	NR	TAG = NEFA =		Glucose = Insulin = GIP =	
Summers, 1998 [36]	2 men 6 women (pre- and postmenopausal) 30.5 (18–55) y 24 (19–30) kg/m^2^ 6 h	932	60 (58 en%)	18 (17 en%)	NR	5.9 67.8 mol%	NR	TAG = NEFA =		Glucose = Insulin =	
Yli-Jokipii, 2001 [32]	10 women (premenopausal) 26.9 ± 2.56 y 18.5–25 kg/m^2^ 6 h	NR	55 g/m^2^ body surface area	17 g/m^2^ body surface area	Palm oil IE palm oil	9 31 mol%	0 0	TAG ↓ NEFA = VLDL-C = CM-C =		Glucose = Insulin =	
Yli-Jokipii, 2003 [27]	2 men 7 women (premenopausal) 24 ± 3 y 21.5 ± 2.5 kg/m^2^ 8 h	NR	55 g/m^2^ body surface area	17 g/m^2^ body surface area	IE Lard Lard	52 69 mol%	11.0 ^2^ 12.5	TAG = ^3^ NEFA = TC =		Glucose = Insulin =	
Berry, 2007 [33]	20 men 28.8 ± 10.3 y 23.2 ± 2.6 kg/m^2^ 6 h	853	50 (53 en%)	14 (15 en%)	Palm oil IE palm oil	7.2 37.2 mol%	3.6 15.2	TAG = TC = LDL-C = HDL-C =	FVIIa = WBC =	Glucose = Insulin =	
Sanders, 2011 [7] Filippou, 2014 [37]	25 men 25 women (premenopausal) ± 24.8 y ± 23.5 kg/m^2^ 8 h	846	50 (53 en%)	20 (22 en%)	Palm olein IE palm olein	9.2 39.1 mol%	0 4.7	TAG = NEFA = TC = ApoB48 =		Glucose = Insulin = C-peptide = GIP ↓ PYY=	IL-6 = IL-8 = TNF-α = E-selectin =
Hall, 2014 [34]	11 men 50 ± 7 y 27.6 ± 3.1 kg/m^2^ 6 h	1047	75 (64 en%)	30 (26 en%)	Palm olein IE palm olein	9.8 45.9 mol%	NR	TAG = ^4^ NEFA = TC =			
Hall, 2017 [28]	12 men 20.5 ± 1.1 y 22.4 ± 2.8 kg/m^2^ 4 h	832	52 56 en%	26 28 en%	PSt/PK IE PSt/PK	36.0 54.7 mol%	24 ^5^ 21	TAG ↑		Glucose = Insulin = GIP = PYY =	

Markers are significantly lower (↓), higher (↑) or not significantly different (=) after intake of fats high in C16:0 *sn*-2 contents compared with fats low in C16:0 *sn*-2 contents. ^1^=% of total fatty acids at *sn*-2. ^2^= 12.5% Lard and 11.0% IE lard was solid at 35 °C, and 8.3% and 6.5% at 40 °C respectively. No values reported for 37 °C [27]. ^3^=iAUC of VLDL-TAG was smaller after lard [27]. ^4^=TAG iAUC of 0 to 4 h after IE palm olein was lower than after palm olein (p=0.024). Chylomicron TAG was lower at 4 h after IE palm olein compared to palm olein (p=0.038) [34]. ^5^= 24% PSt/PK and 21% IE PSt/PK was solid at 35 °C, and 17 and 11% at 40 °C respectively. No values for 37 °C [28]. Abbreviations: apo, apolipoprotein; CM-C, chylomicron cholesterol; en%, % of total energy; FVIIa, activated factor VII; GIP, glucose-dependent insulinotropic polypeptide; HDL-C, high-density lipoprotein cholesterol; IE, interesterified; IL, interleukin; LDL-C, low-density lipoprotein cholesterol; NEFA, non-esterified fatty acids; NR, not reported; PSt/PK, palm stearin blended with palm kernel; PYY, peptide YY; TAG, triacylglycerol; TC, total cholesterol; TNF, tumor necrosis factor; VLDL, very low-density lipoprotein; WBC, white blood cells; wt, weight; y, year.

**Table A5:** Postprandial effects of substituting fats low in stearic acid (C18:0) *sn*-2 contents with fats high in C18:0 *sn*-2 contents on cardiometabolic risk markers.

First author, Year of publication	Population, Age, BMI, Follow-up	Total energy (kcal)	Total fat in grams (en%)	C18:0 content in grams (en%)	Source Low *sn*-2 High *sn*-2	C18:0 *sn*-2 in fat blends (% ^1^)	Solid at 37 °C (%)	Lipids and lipoproteins		Hematological markers	Other markers
Summers, 1999 [29]	14 women 49 (29–70) y 27.5 (20.6–52.8) kg/m^2^ 6 h	932	60 (58 en%)	18 (18 en%)	NR	NR 83.3	NR	TAG = NEFA =			Glucose = Insulin =
Sanders, 2003 [30]	17 men 38.2 ± 11.1 y 24.5 ± 2.9 kg/m^2^ 6 h	749	50 (60 en%)	17 (20 en%)	Cocoa butter IE cocoa butter	NR	NR ^2^	TAG ↓	TC = LDL-C =	FVIIa ↓	
Berry, 2007 [14]	16 men 26.8 ± 8.0 y 23.7 ± 3.7 kg/m^2^ 8 h	853	50 (53 en%)	26 (28 en%)	Native or IE shea butter blended with HOSO	3.1 22.8 mol%	22.2 41.2	TAG = NEFA =	TC = LDL-C = HDL-C =	FVIIa = WBC =	Glucose = Insulin =
Robinson, 2009 [31]	10 non-obese men (55.8 ± 7.0y, 26.6 ± 2.5 kg/m^2^) 11 obese men (59.3 ± 6.0y, 32.9 ± 4.3 kg/m^2^), 6 h	NR	86-102 (76 en%) (1 g/kg body mass)	25-30 (21 en%)	Canola stearin (EIE, CIE, native) blended with HOSO	0.5 0.6 25.5 wt%	5.4 5.6 18.6	Non-obese: TAG = Obese: TAG ↓ ^3^	Both: NEFA = TC = LDL-C = HDL-C		Both: Glucose = Insulin =

Markers are significantly lower (↓), higher (↑) or not significantly different (=) after intake of fats high in C18:0 *sn*-2 contents compared with fats low in C18:0 *sn*-2 contents. ^1^=% of total fatty acids at *sn*-2. ^2^=Melting points were 35 and 50 °C for native and randomized cocoa butter, respectively [30]. ^3^=The native blend (high C18:0 *sn*-2) had a lower TAG response compared to the chemically interesterified blend (low C18:0 *sn*-2) but not compared to the enzymatically interesterified blend [31]. Abbreviations: CIE, chemically interesterified; en%, % of total energy; EIE, enzymatically interesterified; FVIIa, activated factor VII; HDL-C, high-density lipoprotein cholesterol; HOSO, high oleic sunflower oil; IE, interesterified; LDL-C, low-density lipoprotein cholesterol; NEFA, non-esterified fatty acids; NR, not reported; TAG, triacylglycerol; TC, total cholesterol; WBC, white blood cells; wt, weight; y, year.

**Table A6:** Postprandial effects of substituting fats high in palmitic acid (C16:0) with fats high in stearic acid (C18:0) on cardiometabolic risk markers.

First author, year of publication	Population, Age, BMI, Postprandial follow-up	Total energy (kcal)	Total fat in grams (en%)	Content C16:0 C18:0 (g)	Content C16:0 C18:0 (en%)	Source C16:0 C18:0	Lipids and lipoproteins	Hematological markers	Other markers	
Mennen, 1998 [42]	91 women (postmenopausal) 75.7 ± 5.2 y 27.7 ± 4.1 kg/m^2^ 6–7 h	948– 889	55.7–49.3 (53–50 en%)	22 19	21 19	NR	TAG =	FVIIa =		
Jensen, 1999 [38]	15 women (premenopausal) 8 normal-weight (27 ± 2 y, 19.2–23.7 kg/m^2^) 7 overweight (29 ± 3 y, 28.8–47.5 kg/m^2^) 8 h	406kcal/m^2^ body surface area	29 g/m^2^ (65 en%)	12 g/m^2^ 5 g/m^2^	27 10	Palm oil Lard	Both: TAG =		Both: Insulin = Leptin =	
Sanders, 2000 [39]	11 men5 women (premenopausal) 25.5 (18–32) y 23.2 (20.1–27.8) kg/m^2^ 7 h	1242	90 (65 en%)	37 36	27 26	Palm oil Hydrogenated and IE HOSO	TAG =	FVIIa = FVIIc =		
Tholstrup, 2001 [41] + 2003 [44] + 2004 [43]	16 men 23.4 ± 2.4 y 23 ± 2 kg/m^2^ 8 h	1672 ^1^	75 ^1^ (50.6 en%^2^)	32 ^1^ 34 ^1^	17 18	IE blend of tripalmitin or tristearin with HOSO	TAG = NEFA = VLDL-C = LDL-C = HDL-C = ApoB = ApoA1 = Lp[a] =	FVIIa = FVIIc = PAI-1 antigen = tPA activity =	CETP activity = LPL activity =	
Teng, 2011 [40]	10 men 21.9 ± 0.7 y 21.0 ± 1.6 kg/m^2^ 4 h	754	50 (60 en%)	17 9	21 10	Palm olein Lard	TAG ↓ NEFA =		Glucose = Insulin = Leptin =	IL-6 = TNF-α = IL-1ß =
Sanders, 2011 [7] Filippou, 2014 [37]	25 men 25 women (premenopausal) ± 24.8y, ± 23.5 kg/m^2^ 8 h	846	50 (53 en%)	20 9	22 9	Palm olein Lard	TAG ↓ NEFA ↓ TC = ApoB48 =		Glucose = Insulin = C-peptide = GIP ↓ PYY=	IL-6 = IL-8 = TNF-α = E-selectin =

Markers are significantly lower (↓), higher (↑) or not significantly different (=) after intake of fats high in C18:0 compared with fats high in C16:0. ^1^=per 75 kg body weight. Range of fat intake was 65-85 grams [41]. ^2^=50.6 en% was reported. However, our calculations indicate 40.4 en% [41].Abbreviations: apo, apolipoprotein; CETP, cholesteryl ester transfer protein; en%, % of total energy; FVIIa, activated factor VII; FVIIc, Factor VII coagulant activity; GIP, glucose-dependent insulinotropic polypeptide; HDL-C, high-density lipoprotein cholesterol; HOSO, high oleic sunflower oil; IE, interesterified; IL, interleukin; Lp[a], lipoprotein [a]; LPL, lipoprotein lipase; LDL-C, low-density lipoprotein cholesterol; NEFA, non-esterified fatty acids; NR, not reported; PAI, plasminogen activator inhibitor; PYY, peptide YY; TAG, triacylglycerol; TC, total cholesterol; TNF, tumour necrosis factor; tPA, tissue plasminogen activator; VLDL-C, very low-density lipoprotein cholesterol; wt, weight; y, year.
